# Editorial

**Published:** 2013-06-25

**Authors:** F Popa

**Affiliations:** Carol Davila University of Medicine and PharmacyRomania

In our previous editorial, we have underlined the importance of truly understanding the experience of the patient, rethinking the system of improving the healthcare system, while having the patient as the core element, and making a progress by knowing and understanding the reciprocal confidence between the healthcare provider and the patient. As a true evidence of a right understanding of the importance of social change efforts within the framework of the ever-increasing array of health challenges, public health community is rising the use of marketing to design and implement programs to promote the socially beneficial behavior change, including to influence policy makers in adequately addressing the broader social and environmental determinants of health. As we all know, people’s health must be placed in the center of the sustainable development and of adapting the business accordingly, and this also means enhancing public health professionals ‘ability to use social marketing to design public health interventions.

The starting question is “Are we all healthier in the long run?” Because we need to be healthy enough to produce goods and services, including health care services, to have access to them and to have the possibility to buy them. And, as we all know, the economic recession affects all this chain. Within the framework of the patient-public health professionals partnership, the last ones are providing an increasing attention and resources to the right identification of patients segments in order to ensure the necessary priority in public health program development, and to make appropriate resource allocation decisions in the patients’ benefit or to modify the public health programs so as to provide the benefits the patients value most. Our commitment to provide the patients with satisfying exchanges that result in trusting long-term relationships within this mentioned partnership framework is well known.

Taking into account the success of the second edition of the International Congress “Health, Nutrition, Fitness, and Wellbeing – SANABUNAINT 2012” in promoting the Public-Civic-Private Partnership, while considering the entire series of potential benefits (health, social, direct and indirect economic, environmental benefits) - including the fact that “Carol Davila” Academic Publishing House, of “Carol Davila” University of Medicine and Pharmacy in Bucharest, has been awarded The Belgian Prize of Innovation for its contribution in the organization and the successful development of SANABUNA International Congress - we are entitled to say that **SANABUNAINT 2013** will mark a new responsible step in promoting the socially beneficial behavior change at the level of patient-public health professionals partnership by proper community-based activities, by health care training, and skill building combined with the communication activities considering the everyday lives of the patients, their needs, values, and aspirations.

As organizers of **SANABUNAINT 2013** in Autumn, in Bucharest, we are constantly assessing the interaction patterns of the target audience with the educational **SANABUNA** activities, being firmly convinced of the impact of the specified activities on voluntarily adopting healthy behaviors, by creating a large awareness of the benefits of the necessary change, by making clear the alternative choice of “Health, Nutrition, Fitness, and Wellbeing” environment that is more conducive for the necessary socially beneficial behavior change. And, as an expression of the proper evolution of SANABUNA process (planning, research, strategy and program development, implementation and monitoring and so on), by partnering with the patients (we are all patients) in setting the **SANABUNA** agenda so as to create solidarity in building trust and maintain the relationships between stakeholders, and confirming the assumed commitment to break down the “knowledge wall” and to co-operate within the **SANABUNA** intervention to prevent disease and make our lives as patients healthier, while considering all the multiple dimensions of Health and Wellness (as the positive component of good health): social, spiritual, physical, mental, intellectual.

**Fig. 1:**
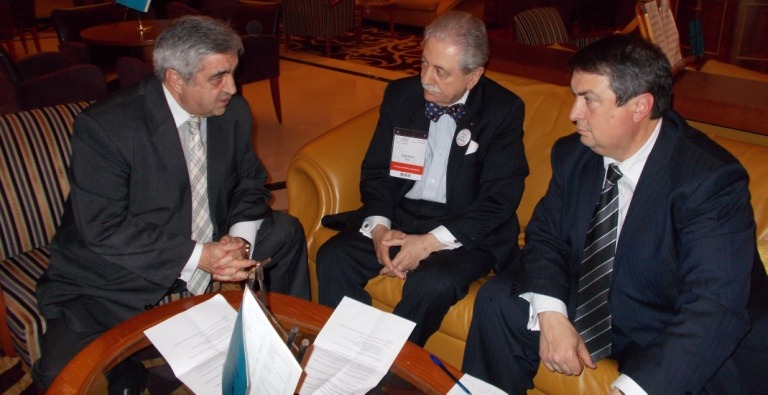
Friendly discussion: Assoc. Prof. Victor Lorin Purcarea, Prof. Eliot Sorel and Prof. Theodor Valentin Purcarea

**Fig. 2:**
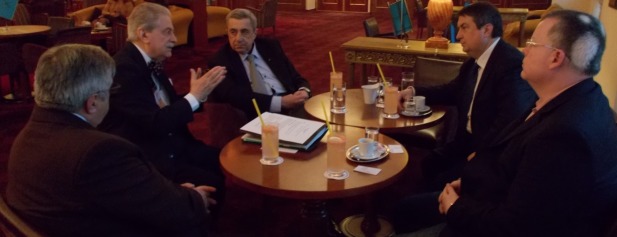
From left to right: Assoc. Prof. Victor Lorin Purcarea, Prof. Eliot Sorel, Adrian Gaspar, Prof. Theodor Valentin Purcarea, Prof. Petru Filip

**Fig. 3:**
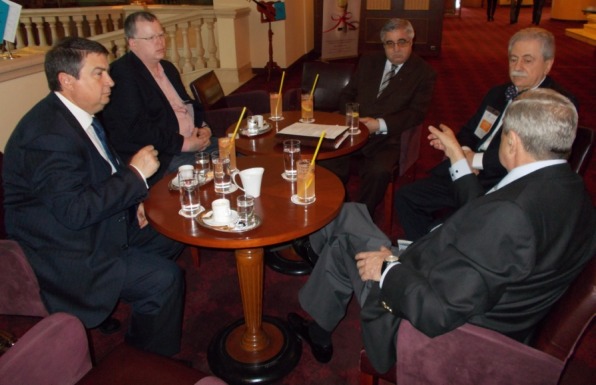
SANABUNA 2013 Working Meeting

That is why we are saluting the **SANABUNA 2013 Working Meeting** from April 12, 2013, at Marriott Grand Hotel Bucharest, attended by **Prof. Florian Popa**, President of SANABUNA International Congress (and Vice-President of the Commission of Public Health of the Romanian Senate), **Prof. Eliot Sorel**, Honorary President of SANABUNA International Congress (MD, DLFAPA, George Washington University, Washington D.C., School of Medicine & School of Public Health, Founder Conflict Management Section WPA, Co-Chair of the Scientific Committee WPA 2013 Bucharest), **Assoc. Prof. Victor Lorin Purcărea**, President of the Organizing Committee of SANABUNA International Congress (Director of “Carol Davila” Academic Publishing House, President of the Commission for Internal and International Policy of the Senate of “Carol Davila” University of Medicine and Pharmacy Bucharest, and Advisor to the Vice-President of the Commission of Public Health of the Romanian Senate), **Adrian Gaşpar**, Member of the Organizing Committee of SANABUNA International Congress (President of Scandia Food, and Member of the Board of the Romanian Distribution Committee), **Prof. Theodor Valentin Purcărea**, Member of the Scientific Committee of SANABUNA International Congress (President of the Romanian Distribution Committee - RDC, Member of the Board of AIDA Brussels, and President of the Executive College of SSMAR). In the context of addressing issues related to the SANABUNA 2013 International Congress, the participants of the Working Meeting have also discussed: a message from **Prof. Petru Filip**, Member of the Scientific Committee of SANABUNA International Congress (Member of the Board of the scientific associations RDC and SSMAR), sent from the USA (being away on an official delegation, as President of the Commission of Foreign Affairs of the Romanian Senate); the Outlook **“Restart Romania 2014-2020”** developed by **Prof. Eliot Sorel**, Honorary President of SANABUNA International Congress.

 Prof. Dr. Florian Popa

